# Effectiveness of Calcium Hydroxide and Gingerols Mixture as a Novel Obturation Material for Infected Root in Primary Teeth: A Randomized Clinical Trial

**DOI:** 10.1155/2024/5528260

**Published:** 2024-02-08

**Authors:** Fathi A. Qasem, Salwa M. Awad, Rizk A. Elagamy

**Affiliations:** ^1^Department of Pediatric Dentistry, Faculty of Dentistry, Thamar University, Thamar, Yemen; ^2^Department of Pediatric Dentistry and Dental Public Health, Faculty of Dentistry, Mansoura University, Mansoura, Egypt

## Abstract

**Introduction:**

The tendency to use dental materials of plant origin is one of the prevailing trends in dentistry to reduce exposure to materials that could have some toxic impact in the long term.

**Objective:**

To evaluate the efficacy of calcium hydroxide combined with gingerols (Ginge-Cal) as a novel obturation material for treating infected primary teeth and decreasing the recurrence of infection.

**Materials and Methods:**

The study was conducted on 30 lower primary molars with infected pulp for children aged 4–8 years. The sample was randomly divided into two groups depending on the tested obturation material: Ginge-Cal group and the Metapex group. The evaluation was done by different parameters clinically and radiographically at various intervals up to 12 months.

**Results:**

Based on chi-squared and McNamara's test with a 5% significance level, the clinical results indicated that Ginge-Cal group was more effective than the Metapex group in reducing or eliminating pain (*P*=0.467) after 1 week, sensitivity to percussion (*P*=0.090) at 3 months of follow-up, purulent swelling (*P*=0.444) at 6 and 9 months of follow-up, fistula, and tooth mobility. The radiographic results, based on the periapical and furcation area radiolucency at 12 months of follow-up, favored Ginge-Cal group over the Metapex group (*P*=0.683), (*P*=0.456), respectively. There were no statistically significant differences in pathological root resorption and periodontal space. The differences within the Ginge-Cal group were directly influenced by the time intervals in a statistically significant manner, ranging from (*P*=0.004) to (*P* < 0.001). The success percentage was 87.5% for Ginge-Cal group and 64.3% for Metapex group.

**Conclusions:**

Ginge-Cal can be considered a promising material for treating the infected root canal when used as an obturation material for the infected root canal. This trial is registered with NCT05181813.

## 1. Introduction

Pulp therapy of necrotic or older failed treated teeth has a high percentage of inflammation recurrence. This is due to the significant microbial diversity in children pulp and tissues around the apex of the root and furcation zone. Additionally, the development of microbial resistance to antibiotics and the difference in microbial presence contribute to this problem [1]. Moreover, most materials are unable to eliminate the microorganisms in this area. Therefore, several mechanical and chemical techniques have been developed to increase the efficiency of the materials in eliminating the pulp canal microbes. These techniques include improving irrigation solutions, intracanal medicaments, or intracanal filling materials [2].

Over time, various intracanal obturation materials have been used to obtain the best results in pulp therapy of primary teeth. Among the most used materials in the treatment of the infected pulp of primary teeth are materials based on calcium hydroxide (Ca(OH)_2_) and Iodoform such as Vitapex and Metapex. Metapex (Meta Biomed Co., Ltd., Korea) is very popular in the Middle East and is very similar to Vitapex®. It is a premixed syringe pulpectomy obturating material composed of calcium hydroxide (Ca(OH)_2_) (<36 wt.%), iodoform (<37 wt.%), and polydimethylsiloxane oil-based (<27 wt.%). It has excellent radiopacity and antibacterial properties. It can reduce pain, inflammation, and pathological resorption in infected teeth. It can also promote bone remodeling and tissue regeneration. However, Metapex may have some limitations, such as poor distribution and weak inhibition of resorption [3].

Recently, several natural products have been studied for possible uses in pulp therapy of primary and permanent teeth [4]. Khairwa et al. [5] evaluated clinically and radiographically a mixture of zinc oxide eugenol and Aloe Vera as a root-filling material. They concluded that a mixture of Aloe Vera gel and zinc oxide powder in primary teeth showed good clinical and radiographic success in endodontic treatment [5]. Kumar et al. [6] evaluated clinically Curcuma powder as a pulpotomy medicament in children teeth. They found that Curcuma powder in the primary teeth pulpotomy treatment had good clinical and radiographic outcomes [6].

In the last few years, more scientific research has focused on ginger mechanisms, targets, and numerous components. Many studies have evaluated the effectiveness of ginger and its ingredients in some medical conditions as an antioxidant, antibacterial agent, antivomiting compound, antiasthma, antinausea compound, and anticancer agent [7, 8]. Moreover, it reduces the incidence of dementia, diabetes, cardiovascular disease, platelet aggregation, ulcerative colitis, and cholesterol. Gingerols are the major powerful phenolic compounds present in ginger and are renowned for their role in human health and nutrition. Through the Gingerol study, different types of Gingerol are homologous and differ in the length of their unbranched alkyl side chain: (6) –Gingerol, (8) –Gingerol, (10) –Gingerol (12) –Gingerol, and (14) –Gingerol [9]. In dentistry, Gingerols were tested in the lab as an antimicrobial agent in a previous study [10].

Park et al. [11] evaluated the antibacterial activity of Gingerols against oral periodontal pathogens. The results showed that (10,12)-Gingerol evidenced potent antibacterial activity in vitro against anaerobic bacteria associated with periodontitis [11]. The mixture of Gingerols with Ca(OH)_2_, called Ginge-Cal, was compared to a against of Metapex in the treatment of inflamed primary teeth in a previous study by Qasem et al. [10, 12]. They found that Ginge-Cal was superior to Metapex in eliminating microorganisms that were cultured and tested in the lab from the inflamed pulp of primary teeth. Based on the reviews and the limited or lack of valuable studies to evaluate Gingerols in dentistry, especially in root canal obturation, and based on Gingerol anti-inflammatory properties, this study focused on the effectiveness of Ginge-Cal in the treatment of primary teeth with infected necrotic pulp versus Metapex clinically and radiographically.

## 2. Materials and Methods

This study follows the rules of the Ethical Committee of the Faculty of Dentistry Mansoura University, Egypt, and medical ethics in dealing with children and parents (Code: 06030718 in 2018). The study protocol complied with the Declaration of Helsinki by the World Medical Association. This study was an interventional clinical trial registered at https://clinicaltrials.gov with Clinical Trials Identifier: NCT05181813.

### 2.1. Sampling and Selection

The sample size was calculated based on the previous study of Khairwa et al. [5] by G ^*∗*^Power software (ver. 3.1.9.7) at power = 90% with *α* error = 0.05 and a two-tailed test. Thirty children aged 4–8 were selected at the Faculty of Dentistry Pediatric Dental Clinics. All children included in this study for pulpectomy in one visit had to meet the following inclusion criteria:

- The cooperation of the child and parents during the treatment plan and the commitment to attend the follow-up appointments.
- Parents signed the consent form to participate in the study.
- Absence of any systemic disease that would preclude pulp therapy, such as in children at high risk of subsequent chronic bacteremia.
- No history of a sensitive reaction to any component of the used materials. Presence of 1/2 to 2/3 of the tooth root or more and the root with signs or symptoms of infected necrotic pulp or chronic abscess, with or without sinus tract, soft tissue swelling, mobility, or tenderness to percussion, internal or external root resorption, or unsuccessful past pulp treatment.

Any child not meeting all inclusion criteria or falls behind the specified dates for a period that affects the results were excluded from this study. The children were randomly assigned to two groups based on the obturating material used, as follows:

- Ginge-Cal group (study group): 16 primary molars were filled with Ginge-Cal pulpectomy past.
- Metapex control group: 16 primary molars were filled with Metapex pulpectomy paste.

The randomization was done alternately, so that cases with odd numbers were filled with Ginge-Cal, while cases with even numbers were filled with Metapex. A four-digit code was given to each patient to blind the sample during the evaluation procedures.

### 2.2. Mixture of Ginge-Cal Preparation

The semioil aqueous liquid of Gingerols was obtained in the Department of Pharmacognosy, Faculty of Pharmacy laboratories. The Gingerols extract, CH powder, and barium sulfate powder were combined on a glass slab with a proportion of 3 : 2 : 1. A sterile syringe like a Metapex syringe was filled with the mixture to make it easier to use for obturating the pulp canal [12].

### 2.3. Procedures

Under local anesthesia, the tooth was isolated with a rubber dam, and an access opening was prepared (even through the old stainless crown (SSC)) to remove the remaining pulp tissue or previous obturation material with files. The root canal of the selected tooth was enlarged by manual K file size 15 and irrigated with 3% sodium hypochlorite (NaOCl). The working length of the pulp canal was determined using both periapical radiography and an electronic apex locator. After that, the root canal was instrumented with an endodontic rotary motor and Pro AF Baby Gold files, with a speed of 300–350 rpm and 2 N torque, as per the manufacturer's instructions. After shaping the canal, it was irrigated with 3% NaOCl and dried using a paper point. Then, the canal was filled with either Ginge-Cal or Metapex, depending on the group. Immediate postoperative X-rays checked the quality of the obturation material. The coronal cavity was filled with glass ionomer cement (GIC) material over the obturation material. Then, SSC was placed and the edge and occlusion fit were examined and cemented with GIC. In cases where teeth have not responded to pulpotomy or have undergone unsuccessful treatments in the past, and are now covered with an SSC, a pulpectomy procedure is employed. This process involves accessing the pulp chamber and canals through the existing SSC, thereby eliminating the need for its removal. Following this, a new pulpectomy and pulp obturation using a material selected based on the group is performed on the tooth. The access point is subsequently sealed using a two-layer filling material. The first layer is made up of GIC, while the final layer consists of amalgam. Three different pediatric dentists evaluated all treated teeth clinically and radiographically (Cohen's kappa = 0.72). The scores of each evaluation were recorded for each pedodontist separately and then averaged for each parameter as a final evaluation.

### 2.4. Clinical Evaluation

The teeth underwent clinical evaluation pretreatment and immediately postoperative, after 1 week, 3, 6, 9, and 12 months. The clinical evaluation criteria included: (a) pain related to the treated molars (presence or absence); (b) sensitivity to percussion (presence or absence); (c) purulent swelling (presence or absence); (d) fistula tract in the surrounding soft tissues (presence or absence); and (e) tooth mobility depends on Tomás et al. [13] scoring system.

### 2.5. Radiographical Evaluation

The teeth were subjected to radiographic evaluation using periapical X-ray and bisecting technique before treatment, immediately after treatment, and at 6- and 12-months posttreatment. This evaluation schedule aligns with the guidelines set forth by the American Academy of Pediatric Dentistry for dental radiographic prescription in children. The findings in periapical X-rays for different time intervals were recorded in a data collection sheet for these criteria.

#### 2.5.1. Evaluation of Periapical or Furcation Area Radiolucency

This is done based on the scoring system described by Mendoza et al. [14] as following:

- Periapical area radiolucency score: 0 = no periapical area radiolucency; 1 = one-third or less of the distance between the primary tooth apex and permanent tooth germ; 2 = two-thirds of the distance between the primary tooth apex and permanent tooth germ; and 3 = three-thirds of the distance between the primary tooth apex and permanent tooth germ.
- Furcation radiolucency score: 0 = no furcation radiolucency; 1 = radiolucency covering less than a third of the furcation; 2 = radiolucency ranging between 1/3 and 2/3 of the furcation size; and 3 = radiolucency over 2/3 of the furcation size but remaining over the permanent tooth germ without affecting the tooth.

#### 2.5.2. Pathological Root Resorption

Assessments were made by comparing the tooth roots to adjacent or contralateral teeth during the pretreatment visit. In subsequent visits, the evaluation was done based on the pre-treatment visit, either by increasing or decreasing pathological root resorption (+/−).

#### 2.5.3. Outcomes at 12 Months (Clinically and Radiographically)

The treated tooth clinical and radiological status was categorized by three outcomes at 12 months after the final restoration based on the prior evaluation parameters:

- Quiescent: The tooth showed some clinical or radiological signs or symptoms of inflammation but did so partially after 12 months of treatment than pretreatment.
- Healed: The tooth showed no clinical or radiological signs or symptoms of inflammation after 12 months.
- Failed: The tooth showed some clinical or radiological signs or symptoms of inflammation but did not improve after 12 months of treatment than pretreatment.

## 3. Statistical Analyses

All data were tabulated and coded. Data were fed to the computer and analyzed using IBM SPSS software package version 20.0. (Armonk, NY: IBM Corp.). The Kolmogorov–Smirnov test or Shapiro–Wilk test was used to verify the normality of distribution quantitative data were described using range minimum (min) and maximum (max), mean and standard deviation (SD). To compare the variables and assess the significance of the obtained results at the 5% level, McNemar test, Wilcoxon signed ranks test, and Chi-square test were used.

## 4. Results

**Figure 1:**
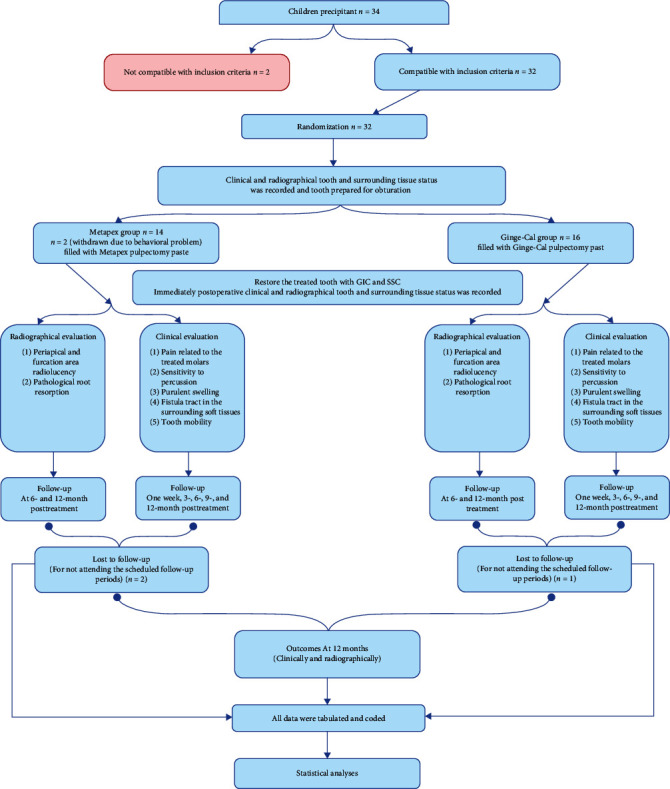
Flowchart for the study.

The treated teeth (*n* = 30) were infected lower primary molars of both genders (Figure 1), 14 boys (46.7%) and 16 girls (53.3%). The children who participated in the study were between 4 and 8 years old, with an average age of 5.87 ± 1.11 years. Three of the treated teeth had to be excluded from the study because of the COVID-19 pandemic and the government's measures to prevent it. The children who had these teeth either did not come back for follow-up visits or did not want to continue the treatment. One of these teeth was in the Ginge-Cal group, which had a drop-out rate of 6.25%, and two were in the Metapex group, which had a drop-out rate of 14.3%.

### 4.1. Clinical Observation Results

The treated teeth were evaluated for five clinical outcomes: pain, sensitivity to percussion, purulent swelling, fistula presence, and tooth mobility. These outcomes were measured at different time intervals after the final restoration: 1 week, 3, 6, 9, and 12 months.

#### 4.1.1. Pain

The comparison between the two studied groups (Ginge-Cal and Metapex) according to pain at different intervals (pretreatment, 1 week, 3, 6, 9, and 12 months). The number and percentage of teeth with absence or presence of pain were reported for each group and interval. The chi-square test and the *P*-value were used to test the statistical significance of the difference between the groups. All teeth had pain before treatment, but most of them became pain-free after 1 week. There was no significant difference (*P*=1.000) between the two groups in terms of pain at any interval. The number of teeth dropped slightly over time due to exfoliation. Only one tooth in each group had pain at 12 months.

#### 4.1.2. Sensitivity to Percussion

The sensitivity to percussion of the two studied groups (Ginge-Cal and Metapex) was compared at different intervals (pretreatment, 1 week, 3, 6, 9, and 12 months). The treated teeth (16 in Ginge-Cal group and 14 in Metapex group) had sensitivity to percussion before treatment, but only 11 in Ginge-Cal group and 11 in Metapex group had it after 1 week. The percentage of teeth without sensitivity to percussion increased over time in both groups, reaching 93.3% in Ginge-Cal group and 75% in Metapex group at 12 months. The only significant difference between the groups was at 3 months, where Ginge-Cal had 100% of teeth without sensitivity to percussion and Metapex had 78.6% (*P*=0.090).

#### 4.1.3. Purulent Swelling

The results for purulent swelling showed that both groups had similar rates of teeth without swelling at all time intervals, except for 1 week and 3 months, where the Ginge-Cal group had higher rates (68.8% vs. 50% and 100% vs. 85.7%, respectively). However, these differences were not statistically significant (*P*=0.414 and *P*=0.090, respectively). At 12 months, almost all of the teeth in both groups did not have purulent swelling (93.3% in Ginge-Cal and 91.7% in Metapex), and this difference was not statistically significant (*P*=1.000). Most teeth had purulent swelling before treatment, but none of them had it after 1 week in Ginge-Cal group and after 3 months in Metapex group. The percentage of teeth without purulent swelling increased over time in both groups, reaching 93.3% in Ginge-Cal group and 91.7% in Metapex group at 12 months. There was a significant reduction in purulent swelling from pretreatment to each follow-up interval in both groups (*P*  < 0.05) (Table 1).

#### 4.1.4. Fistula Presence

The results for fistula presence showed that both groups had similar rates of teeth without fistula at all time intervals, except for pretreatment, where the Ginge-Cal group had a lower rate (43.8% vs. 71.4%). However, this difference was not statistically significant (*P*=0.399). At 12 months, most of the teeth in both groups did not have fistula (93.3% in Ginge-Cal and 83.3% in Metapex), and this difference was not statistically significant (*P*=0.569).

#### 4.1.5. Tooth Mobility

The results for tooth mobility showed that both groups had similar rates of teeth without pathological mobility at all time intervals, except for pretreatment, where the Ginge-Cal group had a higher rate (93.7% vs. 85.7%). However, this difference was not statistically significant. The Ginge-Cal group showed a significant improvement in tooth mobility from 9 to 12 months, while the Metapex group showed a significant improvement from 3 to 12 months. The mobility score of the two groups (Ginge-Cal and Metapex) was compared at different intervals, before treatment, most teeth (10 in Ginge-Cal group and 7 in Metapex group) had a mobility score of 2, but this score was not observed after 6 months in Ginge-Cal group or after 9 months in Metapex group. The percentage of teeth with a mobility score increased over time in both groups, reaching 93.3% in Ginge-Cal group and 75% in Metapex group at 12 months. There was a significant reduction in mobility score from pretreatment to each follow-up interval in Ginge-Cal group (*P*=0.046) (Table 2).

### 4.2. Radiographical Observation Results

The treated teeth were examined for two radiographical outcomes: periapical and furcation area radiolucency and pathological root resorption. Periapical and furcation area radiolucency dark areas around the tooth root or between the roots, indicating bone loss or infection. Pathological root resorption indicates the loss of tooth root structure due to inflammation or infection.

#### 4.2.1. Periapical and Furcation Area Radiolucency

**Figure 2:**
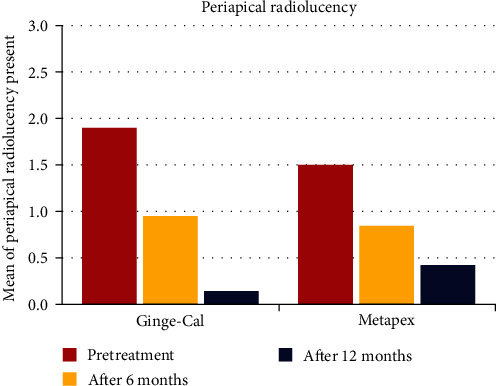
The periapical radiolucency presentation for each group in different studied intervals.

The results for periapical and furcation area radiolucency showed that both groups had similar rates of teeth without radiolucency at all time intervals, except for pretreatment, where the Ginge-Cal group had a higher rate (25% vs. 14.3%). However, this difference was not statistically significant. The Ginge-Cal group showed a significant improvement in radiolucency from 6 to 12 months, while the Metapex group showed a significant improvement from 3 to 12 months. The two groups had a reduction in periapical radiolucency over time, with Ginge-Cal group having a lower mean score than Metapex group at 12 months (0.13 vs. 0.33). The reduction in periapical radiolucency was significant from pretreatment to each follow-up interval in both groups (*P*=0.006) (Table 3 and Figure 2).

**Figure 3:**
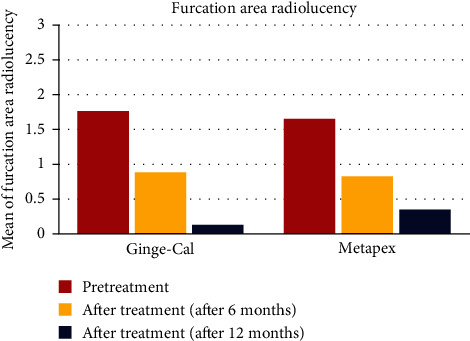
The furcation area radiolucency presentation for each group in different studied intervals.

On another hand, the comparison between the different intervals according to furcation radiolucency is present for two groups. The results indicate that both groups had a reduction in furcation radiolucency over time, with Gingerols group having a lower mean score than Metapex group at 12 months (0.13 vs. 0.42). The reduction in furcation radiolucency was significant from pretreatment to each follow-up interval in both groups (*P*=0.006) (Figure 3).

#### 4.2.2. Pathological Root Resorption

The results for pathological root resorption showed that both groups had similar rates of teeth without resorption at all time intervals, except for pretreatment, where the Ginge-Cal group had a lower rate (25% vs. 35.7%). However, this difference was not statistically significant. The Ginge-Cal group showed a significant improvement in resorption from 6 to 12 months, while the Metapex group showed a significant improvement from 3 to 6 months. Both groups had a decrease in pathologic root resorption over time, with Ginge-Cal group having a higher percentage of teeth with absent resorption than Metapex group at 12 months (93.3% vs. 83.3%). The decrease in pathologic root resorption was significant from pretreatment to each follow-up interval in both groups (*P*  < 0.001 vs. *P*=0.001) (Table 4).

### 4.3. Outcomes at 12 Months (Clinically and Radiographically)

**Figure 4:**
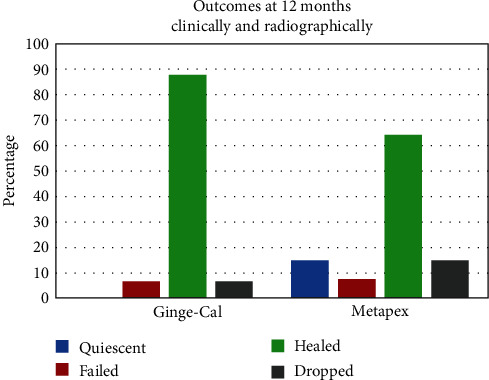
The outcomes at 12 months in two studied groups (clinically and radiographically).

**Figure 5:**
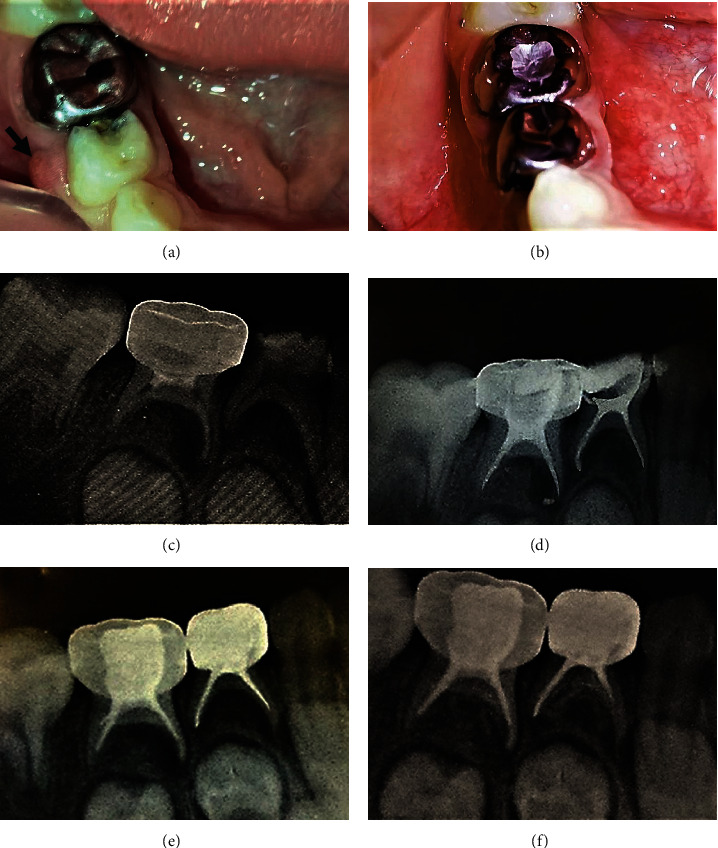
Molars (84 (an infected tooth with discharge fistula)—85 (previous failed pulpotomies tooth)) of a 6.5-year-old girl were treated with Ginge-Cal obturation material. (a) Pretreatment photo the black arrow indicates to fistula. (b) After 12 months of obturation photo (c) pretreatment X-ray, (d) postobturation X-ray, (e) after 6 months of obturation X-ray, and (f) after 12 months of obturation X-ray.

The main comparison between the two groups according to pulpectomy outcomes at 12 months. The Ginge-Cal group had a higher percentage of healed teeth than Metapex group (87.5% vs. 64.3%), while Metapex group had a higher percentage of quiescent than Ginge-Cal group (14.3% vs. 0%). The failed and dropped percentages were similar in both groups (6.25% vs. 7.1% and 6.25% vs. 14.3%, respectively). The comparison between the groups was not statistically significant (*P*=0.441) (Figures 4 and 5).

## 5. Discussion

Due to limited knowledge of any previous study about Gingerols in dental treatment. Also, there is a lack of previous clinical studies about a mix of Gingerols or Ginger oil with Ca(OH)_2_. Therefore, this study is considered the first study that discussed the topic of children infected teeth.

The results of clinical observation showed a decrease in the pain and the sensitivity to percussion in the treated tooth by Ginge-Cal faster than in teeth treated by Metapex with no statistically significant difference. The clinical observation results showed that Ginge-Cal reduced pain and sensitivity to percussion faster than Metapex, although the difference was not statistically significant. However, there was a significant difference within each group when comparing the materials at different intervals, with Ginge-Cal showing superior performance in all follow-up periods. One week after treatment, there was no significant difference between the two materials in terms of sensitivity to percussion. This may be attributed to the pain-relieving and anti-infective properties of Gingerols, which reduce the inflammation of infected tissues by decreasing the microbial and inflammatory cell accumulation, as supported by previous studies [12, 15, 16]. The combination of Ca(OH)_2_ and Gingerols has a dual action that enhances this effect. Metapex also reduced pain, possibly due to the presence of Iodoform as a sedative and Ca(OH)_2_ as an anti-inflammatory agent in its composition, as confirmed by Singh et al. [17].

The results of this study also showed that Ginge-Cal was more effective than Metapex in treating purulent swelling and fistula formation, with a shorter time and no significant difference. This could be explained by the antimicrobial, anti-inflammatory, and antioxidant properties of both Gingerols and Ca(OH)_2_. It could also be due to the hydroxyl ions that create a highly alkaline environment that destroys lipids, leading to structural damage to bacterial proteins and nucleic acids. Calcium hydroxide can also activate tissue enzymes that promote tissue regeneration [18]. Gingerols enhance this effect with their antimicrobial, anti-inflammatory, and antioxidant properties. These findings are consistent with some previous studies [12, 19–21]. Regarding tooth mobility, Metapex has been shown to reduce it effectively in previous studies, according to Gupta and Das [22] and Abo et al. [23], due to its anti-inflammatory effect on the periodontal ligament (PDL) space. Ginge-Cal was superior in its results, possibly because it reduced the inflammation and PDL space faster, as consistent with the previous experimental study [12]. This could be attributed to the anti-inflammatory effect of both materials on the PDL, as established by Mortazavi and Baharvand [24] and Siddiqui et al. [25]. Moreover, ginger has an antiosteoclastogenic effect that prevents bone resorption in periodontitis, as confirmed by Saad [26] and Kim et al. [27]. They suggested the potential use of ginger as an antiresorptive strategy in periodontitis.

The results of this study showed that Ginge-Cal was better than Metapex in reducing periapical and furcation radiolucency and pathological resorption, although the difference was not statistically significant. This could be explained by the anti-inflammatory effect and the bone remodeling activity of Ginge-Cal, as observed in the histopathological slides of a previous study [12]. The efficacy of Gingerols on bone was also demonstrated by Fan et al. [28] who concluded that Gingerols is a promising material for treating bone destruction and enhancing bone remodeling.

Regarding pathological root resorption, the results showed that both materials decreased it, but Ginge-Cal had a higher percentage of inhibition than Metapex, with a significant difference at different intervals. This has not been reported by previous studies to our knowledge. Gupta et al. [29] studied pathological resorption with Metapex and found a relative weakness in its prevention. This may be due to the poor distribution of Metapex in the treatment area, which Ginge-Cal achieves more effectively, due to its high flow.

The microscopic bone remodeling ability of the two materials may be attributed to the presence of Ca(OH)_2_. Nascimento et al. [30] and De Souza et al. [31] highlighted the role of Ca(OH)_2_ as an antibone destruction material. These results are in agreement with this study. Ginge-Cal was superior to Metapex, possibly because of its antioxidant effect, which plays an important role in bone remodeling. These findings and explanations are consistent with Domazetovic et al. [32] who concluded that antioxidants help in the bone healing process.

The study had some limitations, such as the use of scores and descriptions instead of computerized digital radiographs, which would have allowed for more precise comparisons. The COVID-19 pandemic, precautionary measures, and the repeated closure of college clinics limited the follow-up time for clinical cases. Furthermore, some parents did not adhere to the scheduled dates for follow-up, which required the re-selection of cases several times.

## 6. Conclusions

In the presence of some limitations, the Ginge-Cal is better than Metapex for treating preapical infection. Also, the Ginge-Cal decreases bone destruction and enhances bone remodeling more than Metapex. Ginge-Cal can be considered a promising material in treating the infected root canal as an obturation material or as an intercanal medicament for the infected root canal. Gingerols is an opportunity for success if used in endodontic material in general.

## Figures and Tables

**Table 1:** Comparison at different studied intervals according to purulent swelling for two groups.

Purulent swelling	Pretreatment		Follow-up intervals									
			1 week		3 months		6 months		9 months		12 months	
	No	(%)	No	(%)	No	(%)	No	(%)	No	(%)	No	(%)
Ginge-Cal	(*n* = 16)		(*n* = 16)		(*n* = 16)		(*n* = 15^#^)		(*n* = 15^#^)		(*n* = 15^#^)	
Absent	3	18.8	16	100	16	100	15	100	15	100	14	93.3
Present	13	81.3	0	0.0	0	0.0	0	0.0	0	0.0	1.0	6.7
*P* ^Mc^			<0.001 ^*∗*^		<0.001 ^*∗*^		<0.001 ^*∗*^		<0.001 ^*∗*^		0.001 ^*∗*^	
Metapex	(*n* = 14)		(*n* = 14)		(*n* = 14)		(*n* = 12^#^)		(*n* = 12^#^)		(*n* = 12^#^)	
Absent	2	14.3	13	92.9	14	100	11	91.7	11	91.7	11	91.7
Present	12	85.7	1	7.1	0	0.0	1	8.3	1	8.3	1	8.3
*P* ^Mc^			0.001 ^*∗*^		<0.001 ^*∗*^		0.004 ^*∗*^		0.004 ^*∗*^		0.004 ^*∗*^	

*P* ^Mc^, *P*-value for McNemar test to compare pretreatment and the different studied intervals in each group; ^#^dropping; and  ^*∗*^statistically significant at *P* ≤ 0.05.

**Table 2:** Comparison at different studied intervals according to mobility score.

Mobility score		Follow-up intervals											
		Pretreatment		1 week		3 months		6 months		9 months		12 months	
		No	(%)	No	(%)	No	(%)	No	(%)	No	(%)	No	(%)
Ginge-Cal		(*n* = 16)		(*n* = 16)		(*n* = 16)		(*n* = 15^#^)		(*n* = 15^#^)		(*n* = 15^#^)	
0		1	6.3	1	6.3	6	37.5	13	86.7	14	93.3	14	93.3
1		5	31.3	7	43.8	10	62.5	2	13.3	0	0.0	0	0.0
2		10	62.5	8	50.0	0	0.0	0	0.0	1	6.7	1	6.7
3		0	0.0	0	0.0	0	0.0	0	0.0	0	0.0	0	0.0
*P* ^Mc^				0.157		0.001 ^*∗*^		0.001 ^*∗*^		0.001 ^*∗*^		0.001 ^*∗*^	
Metapex		(*n* = 14)		(*n* = 14)		(*n* = 14)		(*n* = 12^#^)		(*n* = 12^#^)		(*n* = 12^#^)	
0		2	14.3	1	7.1	4	28.6	7	58.3	9	75.0	9	75.0
1		5	35.7	4	28.6	8	57.1	3	25.0	1	8.3	1	8.3
2		7	50.0	9	64.3	2	14.3	2	16.7	2	16.7	2	16.7
3		0	0.0	0	0.0	0	0.0	0	0.0	0	0.0	0	0.0
*p* ^Mc^				0.450		0.052		0.099		0.046 ^*∗*^		0.046 ^*∗*^	

*P* ^Mc^, *P*-value for McNemar test to compare pretreatment and the different studied intervals in each group;  ^*∗*^statistically significant at *P* ≤ 0.05; and ^#^dropping.

**Table 3:** Comparison between the different studied intervals according to periapical radiolucency present for each group.

Periapical radiolucency present	Pretreatment	Follow-up intervals	
		6 months	12 months
Ginge-Cal	(*n* = 16)	(*n* = 15^#^)	(*n* = 15^#^)
Min.–max.	1.0–3.0	0.0–2.0	0.0–2.0
Mean ± SD	1.75 ± 0.58	0.87 ± 0.52	0.13 ± 0.52
*P*	—	0.001 ^*∗*^	<0.001 ^*∗*^
Metapex	(*n* = 14)	(*n* = 12^#^)	(*n* = 12^#^)
Min.–max.	1.0–2.0	0.0–2.0	0.0–2.0
Mean ± SD	1.64 ± 0.50	0.83 ± 0.72	0.33 ± 0.78
*P*	—	0.013 ^*∗*^	0.006 ^*∗*^

*P*, *P*-value for Wilcoxon signed ranks test for comparing pretreatment and the different studied intervals in each group;  ^*∗*^statistically significant at *P* ≤ 0.05; and ^#^dropping.

**Table 4:** Comparison between the different studied intervals according to pathologic root resorption in two groups.

Pathologic root resorption	Pretreatment		Follow-up intervals			
			6 months		12 months	
	No	(%)	No	(%)	No	(%)
Ginge-Cal	(*n* = 16)		(*n* = 15^#^)		(*n* = 15^#^)	
Absent	4	25.0	4	26.7	14	93.3
Present	12	75.0	0	0.0	0	0.0
Increase	0	0.0	1	6.7	1	6.7
Decrease	0	0.0	10	66.7	0	0.0
*P*	—		<0.001 ^*∗*^		<0.001 ^*∗*^	
Metapex	(*n* = 14)		(*n* = 12^#^)		(*n* = 12^#^)	
Absent	5	35.7	5	41.7	10	83.3
Present	9	64.3	0	0.0	0	0.0
Increase	0	0.0	1	8.3	1	8.3
Decrease	0	0.0	6	50.0	1	8.3
*P*	—		<0.001 ^*∗*^		0.001 ^*∗*^	

*P*, *P*-value for Chi-square test for compare pretreatment and studied intervals in each group;  ^*∗*^statistically significant at *P* ≤ 0.05; and ^#^dropping.

## Data Availability

Data are available upon request from the corresponding author.

## References

[1] Brustolin J. P., Mariath A. A. S., Ardenghi T. M., Casagrande L. (2017). Survival and factors associated with failure of pulpectomies performed in primary teeth by dental students. *Brazilian Dental Journal*.

[2] Chonat A., Rajamani T., Rena E. (2018). Obturating materials in primary teeth—a review. *Research & Reviews: Journal of Dental Sciences*.

[3] Cwikla S. J., Bélanger M., Giguère S., Progulske-fox A., Vertucci F. J. (2005). Dentinal tubule disinfection using three calcium hydroxide formulations. *Journal of Endodontics*.

[4] Almadi E. M., Almohaimede A. A. (2018). Natural products in endodontics. *Saudi Medical Journal*.

[5] Khairwa A., Bhat M., Sharma R., Satish V., Maganur P., Goyal A. (2014). Clinical and radiographic evaluation of zinc oxide with aloe vera as an obturating material in pulpectomy: an in vivo study. *Journal of Indian Society of Pedodontics and Preventive Dentistry*.

[6] Kumar R., Purohit R. N., Bhatt M., Purohit K., Acharya J. (2017). Clinical and radiological evaluation of turmeric powder as a pulpotomy medicament in primary teeth: an in vivo study. *International Journal of Clinical Pediatric Dentistry*.

[7] Malu S. P., Obochi G. O., Tawo E. N., Nyong B. E. (2009). Antibacterial activity and medicinal properties of ginger (*Zingiber officinale*). *Global Journal of Pure and Applied Sciences*.

[8] Kumara M., Shylajab M., Nazeemc P., Babu T. (2017). 6-gingerol is the most potent anticancerous compound in ginger (Zingiber officinale Rosc.). *Journal of Developing Drugs*.

[9] Mao Q.-Q., Xu X.-Y., Cao S.-Y. (2019). Bioactive compounds and bioactivities of ginger (Zingiber officinale Roscoe). *Foods*.

[10] Qasem F. A., Awad S. M., ELagamy R. A. (2022). Anti-microbial efficacy of gingerols and calcium hydroxide mixture versus metapex on microorganism of infected root canal of primary teeth. *EC Dental Science*.

[11] Park M., Bae J., Lee D. S. (2008). Antibacterial activity of [10]-gingerol and [12]-gingerol isolated from ginger rhizome against periodontal bacteria. *Phytotherapy Research*.

[12] Qasem F. A., Awad S. M., ELagamy R. A., Badria F., Al-Gabri N. (2022). Histopathological evaluation of calcium hydroxide mixed with gingerols versus metapex as obturating material in treatment of infected pulp in primary teeth. *International Journal of Science and Research Methodology*.

[13] Tomás I., Álvarez M., Limeres J., Potel C., Medina J., Diz P. (2007). Prevalence, duration and aetiology of bacteraemia following dental extractions. *Oral Diseases*.

[14] Mendoza A., Reina J. E. S., Garcia-Godoy F. (2010). Evolution and prognosis of necrotic primary teeth after pulpectomy. *American Journal of Dentistry*.

[15] Thomson M., Al-Qattan K. K., Al-Sawan S. M., Alnaqeeb M. A., Khan I., Ali M. (2002). The use of ginger (Zingiber officinale Rosc.) as a potential anti-inflammatory and antithrombotic agent. *Prostaglandins, Leukotrienes and Essential Fatty Acids*.

[16] Aryaeian N., Tavakkoli H. (2015). Ginger and its effects on inflammatory diseases. *Advances in Food Technology and Nutritional Sciences—Open Journal*.

[17] Singh V., Das S., Sharma N. K. (2012). Iodoform: a boon in disguise. *Open Journal of Stomatology*.

[18] Tamburić S. D., Vuleta G. M., Ognjanović J. M. (1993). In vitro release of calcium and hydroxyl ions from two types of calcium hydroxide preparation. *International Endodontic Journal*.

[19] Mashhadi N. S., Ghiasvand R., Askari G., Hariri M., Darvishi L., Mofid M. R. (2013). Anti-oxidative and anti-inflammatory effects of ginger in health and physical activity: review of current evidence. *International Journal of Preventive Medicine*.

[20] Ricci C., Travert V. Calcium hydroxide in endodontics. *Revue Francaise Dendodontie Publication Officielle de La Societe Francaise Dendodontie*.

[21] McHugh J. (2021). Getting to the root of the anti-inflammatory effects of ginger. *Nature Reviews Rheumatology*.

[22] Gupta S., Das G. (2011). Clinical and radiographic evaluation of zinc oxide eugenol and metapex in root canal treatment of primary teeth. *Journal of Indian Society of Pedodontics and Preventive Dentistry*.

[23] Abo A., Elbadrawy B., Aldeen A., Allah A., Elhamed A., Eisa A. E. Y. (2021). Clinical and radiographic assessment of Turmeric and Aloe barbadensis mix as a capping material in pulpotomized primary teeth. *Al-Azhar Journal of Dental Science*.

[24] Mortazavi H., Baharvand M. (2016). Review of common conditions associated with periodontal ligament widening. *Imaging Science in Dentistry*.

[25] Siddiqui Y. D., Omori K., Ito T. (2019). Resolvin D2 induces resolution of periapical inflammation and promotes healing of periapical lesions in rat periapical periodotitis. *Frontiers in Immunology*.

[26] Saad F. A. (2019). The possible therapeutic role of ginger extract in the effect of chronic aluminum toxicity on rat periodontium (histological and immunohistochemical study). *Egyptian Journal of Histology*.

[27] Kim Y.-G., Kim M. O., Kim S.-H. (2020). 6-Shogaol, an active ingredient of ginger, inhibits osteoclastogenesis and alveolar bone resorption in ligature-induced periodontitis in mice. *Journal of Periodontology*.

[28] Fan J. Z., Yang X., Bi Z. G. (2015). The effects of 6-gingerol on proliferation, differentiation, and maturation of osteoblast-like MG-63 cells. *Brazilian Journal of Medical and Biological Research*.

[29] Gupta B., Singh I., Goyal P., Garg S., Gupta S. (2019). A clinical and radiographic study of four different root canal filling materials in primary molars—an in vivo study. *Dental Journal of Advance Studies*.

[30] Nascimento C. D., Paulo J., Issa M. (2008). Bone repair using mineral trioxide aggregate combined to a material carrier, associated or not with calcium hydroxide in bone defects. *Micron*.

[31] De Souza R. S., De Souza V., Holland R., Gomes-Filho J. E., Murata S. S., Sonoda C. K. (2009). Effect of calcium hydroxide-based materials on periapical tissue healing and orthodontic root resorption of endodontically treated teeth in dogs. *Dental Traumatology*.

[32] Domazetovic V., Marcucci G., Iantomasi T., Brandi M. L., Vincenzini M. T. (2017). Oxidative stress in bone remodeling: role of antioxidants. *Clinical Cases in Mineral and Bone Metabolism*.

